# Sequence Types, Clonotypes, Serotypes, and Virotypes of Extended-Spectrum β-Lactamase-Producing *Escherichia coli* Causing Bacteraemia in a Spanish Hospital Over a 12-Year Period (2000 to 2011)

**DOI:** 10.3389/fmicb.2019.01530

**Published:** 2019-07-16

**Authors:** Rosalia Mamani, Saskia Camille Flament-Simon, Vanesa García, Azucena Mora, María Pilar Alonso, Cecilia López, Isidro García-Meniño, Dafne Díaz-Jiménez, Jesús E. Blanco, Miguel Blanco, Jorge Blanco

**Affiliations:** ^1^Laboratorio de Referencia de E. coli, Departamento de Microbioloxía e Parasitoloxía, Facultade de Veterinaria, Universidade de Santiago de Compostela, Lugo, Spain; ^2^Unidade de Microbioloxía Clínica, Hospital Universitario Lucus Augusti, Lugo, Spain

**Keywords:** clonotypes, *E. coli*, ESBL, ExPEC, *H*30Rx subclone, ST131 clonal group

## Abstract

The aim of the present study was to examine the prevalence and determine the molecular characteristics of extended-spectrum β-lactamase-producing *Escherichia coli* (ESBL-EC) causing bacteraemia in a Spanish Hospital over a 12-year period (2000 to 2011). As far as we know, this is the first study which has investigated and compared the serotypes, phylogroups, clonotypes, virotypes, and PFGE profiles of ST131 and non-ST131 clones of bacteraemia ESBL-EC isolates. Of the 2,427 *E. coli* bloodstream isolates, 96 (4.0%) were positive for ESBL production: 40 for CTX-M-15, 36 for CTX-M-14, eight for CTX-M-1, four for CTX-M-9, CTX-M-32, and SHV-12. The number of ESBL-EC increased from 1.0% during 2000 to 2005 to 5.5% during 2006–2011 (*P* < 0.001). The 96 ESBL-EC isolates belonged to 36 different STs. The commonest was ST131 (41 isolates), followed by ST58, ST354, ST393 and ST405 (four isolates each). Most CTX-M-15 isolates (87.5%, 35/40) were ST131, whereas the 36 CTX-M-14 isolates belonged to 23 different STs and only 3 (8.3%) of them were ST131. The 35 ST131 CTX-M-15-producing isolates belonged to the *H*30Rx subclone and 29 of them showed the virotype A. A drastic change in ST131 virotypes happened in 2011 due to the emergence of the virotypes E (*sat*, *papGII*, *cnf1*, *hlyA*, and *kpsMII-K5*) and F (*sat*, *papGII*, and *kpsMII-K5*) which displaced virotype A (*afa/draBC*, *afa* operon FM955459, *sat*, and *kpsMII-K2*). Although the 96 ESBL-EC isolates showed 21 O serogroups and 17 H flagellar antigens, 39 belonged to serotype O25b:H4 (ST131 isolates). The second most prevalent serotype (O15:H1) was found to be associated with another important high-risk clone (ST393). In conclusion, the ST131 was the most frequent sequence type, being the *H*30Rx subclone responsible for the significant increase of ESBL-EC isolates since 2006. Here, we report two new virotypes (E and F) of the *H*30Rx subclone emerged in 2011. Future molecular studies are needed to understand the dynamics of expansion of this successful high-risk subclone in order to prevent its spread and establish the importance of the two new virotypes.

## Introduction

*Escherichia coli* is the main cause of bloodstream infections (BSIs) and in recent years there has been a very significant increase in the number of infections caused by multidrug-resistant isolates, and especially by extended-spectrum β-lactamase-producing *E. coli* (ESBL-EC) (Tumbarello et al., 2010; de Kraker et al., 2013; Vihta et al., 2018). ESBL production in *E. coli* has increased due mainly to the spread of CTX-M enzymes. The high-risk clones have played a very important role in the current pandemic spread, especially the ST131 clone associated with the CTX-M-15 enzyme (Nicolas-Chanoine et al., 2008; Chung et al., 2012; Peirano et al., 2012; Adams-Sapper et al., 2013; Banerjee et al., 2013; Colpan et al., 2013; Matsumura et al., 2013; Johnson et al., 2017; Kallonen et al., 2017; Roer et al., 2017; Mendis et al., 2018).

The majority of ST131 isolates belong to the O25b:H4 serotype, subclone *H*30, clonotype CH40-30 and clade C. The *H*30Rx (clade C2) subset often carries *bla*_CTX–M–15_, whereas *H*30R-M27 (clade C1-M27) subset is positive for *bla*_CTX–M–27_ and *H*30R-nM27 (clade C1-nM27) subset is often positive for *bla*_CTX–M–14_. However, there are other two ST131 subclones less frequently expanded: *H*22 (clonotype CH40-22, clade B) and *H*41 (clonotype CH40-41, clade A). Isolates of subclone *H*41 usually belong to serotype O16:H5 (Price et al., 2013; Olesen et al., 2014; Peirano et al., 2014; Matsumura et al., 2017a,b; Harris et al., 2018). Generally, clades A and B show fluoroquinolone (FQ) susceptibility and rarely carry ESBL plasmids, while most isolates of clade C are FQ-resistant. Clade B evolved into clade C by acquisition of several prophages, genomic islands, the *fimH*30 allele and mutations within *gyrA a*nd *parC* genes, mainly during the late 1980s (Ben Zakour et al., 2016; McNally et al., 2016; Stoesser et al., 2016; Pitout and DeVinney, 2017). In addition, considering the content of virulence genes, ST131 isolates can be classified into 12 virotypes (A to F) (Blanco et al., 2013; Dahbi et al., 2014; Mora et al., 2014).

Currently, few data are available on molecular epidemiology of bacteraemia caused by ST131 and non-ST131 clones of ESBL-EC, especially in Europe (Rodríguez-Baño et al., 2012; Horner et al., 2014; Day et al., 2016; Merino et al., 2016; Roer et al., 2017). Thus, the aim of the present study was to examine the prevalence and determine the molecular characteristics of ESBL-EC causing bacteraemia in a Spanish Hospital over a 12-year period (2000–2011).

## Materials and Methods

### *E. coli* Bloodstream Isolates

The present study included 2,427 non-duplicate, clinically relevant *E. coli* isolates recovered from blood of patients at Hospital Universitario Lucus Augusti (HULA) (formely Complejo Hospitalario Xeral-Calde) between January 2000 and December 2011. All available isolates from that time period were analyzed. HULA is a university hospital with 740 beds, which serves an urban and rural population of approximately 265,000 inhabitants in Lugo, Spain. Ethics approval was not required according to national and institutional guidelines.

### Antibiotic Susceptibility Testing and ESBL Typing

ESBL production was detected by the double disk synergy test (Jarlier et al., 1988). The type of ESBL was established by PCR and sequencing using the TEM-, SHV-, CTX-M-1, and CTX-M-9 group-specific primers (Leflon-Guibout et al., 2004; Blanco et al., 2009).

### Phylogenetic Grouping, MLST, CH Typing, and Identification of *H*30Rx Subclone

The determination of the phylogenetic groups (A, B1, B2, C, D, E, F) was carried out by the protocol of Clermont et al. (2013). The sequence types (STs) were established following the multilocus sequence typing (MLST) scheme of Achtman^1^ (Wirth et al., 2006). Clonotype identification was determined by *fumC* and *fimH* (CH) sequencing (Weissman et al., 2012; Tchesnokova et al., 2013). The *H*30Rx subclone was established by PCR detection of a specific SNP (G723A) of *ybbW* gene (Banerjee et al., 2013).

### O and H Typing

The serotyping was carried out using all available O (O1 to O181) and H (H1 to H56) antisera (Guinée et al., 1981). Isolates that did not react with any antisera were designated as ONT or HNT (NT, non-typeable) and those non motile were denoted as HNM. The O25b subtype was determined by PCR (Clermont et al., 2008).

### Virulence Genotyping

Virulence genes were screened by PCR as documented previously (Dahbi et al., 2014), using specific primers (Supplementary Table S1). The virulence gene score was the number of extraintestinal virulence-associated genes detected. Isolates were defined presumptively as extraintestinal pathogenic *E. coli* (ExPEC) (Johnson et al., 2015) if positive for ≥2 of 5 markers, including *papAH* and/or *papC*, *sfa/focDE*, *afa/draBC*, *kpsM II*, and *iutA*, and as uropathogenic *E. coli* (UPEC) (Spurbeck et al., 2012) if positive for ≥3 of 4 markers, including *chuA*, *fyuA*, *vat*, and *yfcV*. The virotypes A to F of the ST131 isolates was assigned according to the scheme developed by Dahbi et al. (2014).

### Pulsed Field Gel Electrophoresis (PFGE)

*XbaI*-PFGE profiles were determined as previously described (Mora et al., 2009, 2010) and imported into BioNumerics (Applied Maths, Sint-Martens-Latem, Belgium) and clustered using the UPGMA algorithm and applying Dice coefficient and 1% tolerance.

### Statistical Analysis

All the *P*-values were calculated using the Fisher’s exact test, except for the comparison of the means that was performed using the test one-way ANOVA test. *P*-values <0.05 were considered statistically significant.

## Results

### Prevalence of ESBL-Producing *E. coli* (ESBL-EC)

**FIGURE 1:**
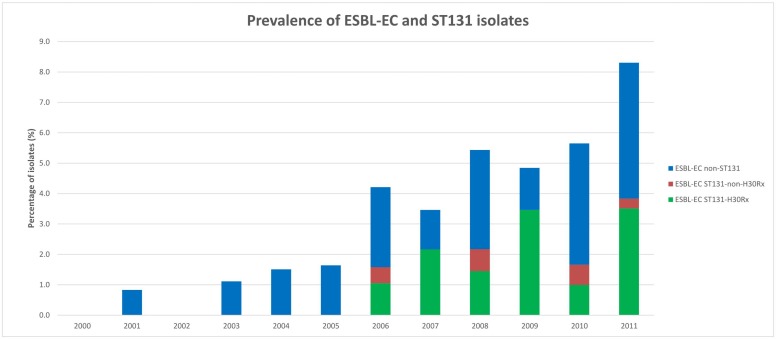
Prevalence of extended-spectrum β-lactamase-producing *E. coli* (ESBL-EC) and ESBL-EC ST131 isolates. A total 2,427 *E. coli* bloodstream isolates were analyzed, of which 96 (4.0%) exhibited ESBL production. A total of 41 ESBL-EC ST131 isolates were identified and 35 of them belonged to the *H*30Rx subclone.

During 2000–2011, 2,427 *E. coli* bloodstream isolates were analyzed, of which 96 (4.0%) exhibited ESBL production. The number of ESBL-EC isolates increased from 1.0% (8/827) during 2000–2005 to 5.5% (88/1,600) during 2006–2011 (*P* < 0.001) (Figure 1, Table 1, and Supplementary Table S2).

**TABLE 1:** Prevalence, serotypes, phylogenetic groups, STs, and clonotypes of extended-spectrum β-lactamase-producing *E. coli* (ESBL-EC) bloodstream isolates.

**Year**	**Number of *E. coli* isolates**	**Number of ESBL isolates (%)**	**Type of ESBL (number of isolates)**	**Serotypes, phylogenetic groups, sequence types, and clonotypes (number of isolates)^a^**
2000	142	0		
2001	120	1 (0.8%)	CTX-M-14 (1)	O2:H5-B2-ST352-CH96-9 (1)
2002	93	0		
2003	90	1 (1.1%)	CTX-M-14 (1)	O15:H1-E-ST393-CH106-54 (1)
2004	199	3 (1.5%)	CTX-M-14 (1)	O105:H21-B1-ST359-CH41-35 (1)
			CTX-M-9 (1)	O144:HNM-B1-ST602-CH19-86 (1)
			CTX-M-1 (1)	O15:HNM-E-ST362-CH100-96 (1)
2005	183	3 (1.6%)	CTX-M-32 (2)	O25a:H25-B1-ST359-CH41-55 (1); O25a:HNM-F-ST648-CH4-58 (1)
			CTX-M-14 (1)	O8:HNT-A-ST48-CH11-0 (1)
2006	190	8 (4.2%)	CTX-M-15 (2)	**O25b:H4-B2-ST131-CH40-30 (2)**
			CTX-M-14 (5)	O8:H7-B1-ST1642-CH4-31 (1); O23:HNM-B1-ST453-CH6-31 (1); **O25b:H4-B2-ST131-CH40-negative (1)**; O102:H6-E-ST405-CH37-27 (1); O153:H34-F-ST354-CH88-58 (1)
			CTX-M-32 (1)	O2:H4-B2-ST95-CH38-27 (1)
2007	231	8 (3.5%)	CTX-M-15 (5)	**O25b:H4-B2-ST131-CH40-30 (5)**
			CTX-M-14 (1)	ONT:H4-A-NEW ST^b^-CH11-25 (1)
			CTX-M-9 (1)	ONT:H10-B1-ST711-CH6-289 (1)
			CTX-M-1 (1)	O8:HNT-B1-ST345-CH4-31 (1)
2008	276	15 (5.4%)	CTX-M-15 (6)	O20:H9-C-ST410-CH4-24 (1); O20:H30-B1-ST156-CH29-38 (1); **O25b:H4-B2-ST131-CH40-30 (4)**
			CTX-M-14 (7)	ONT:HNM-B1-ST58-CH4-32 (1); ONT:H25-B1-ST58-CH4-32 (1); ONT:HNM-C-ST23-CH4-35 (1); ONT:H51-B1-ST359-CH41-35 (1); **ONT:H5-B2-ST131-CH40-41 (1)**; O1:HNM-F-ST354-CH88-58 (1); ONT:H18-D-ST69-CH35-27 (1)
			CTX-M-9 (1)	**O25b:H4-B2-ST131-CH40-22 (1)**
			CTX-M-1 (1)	ONT:H16-B1-ST2602-CH95-38 (1)
2009	289	14 (4.8%)	CTX-M-15 (10)	**O25b:H4-B2-ST131-CH40-30 (10)**
			CTX-M-14 (3)	O15:H1-E-ST393-CH106-54 (3)
			CTX-M-32 (1)	O101:H10-A-ST10-CH11-54 (1)
2010	301	17 (5.6%)	CTX-M-15 (6)	**O25b:H4-B2-ST131-CH40-30 (3)**; O8:H6-E-ST405-CH37-27 (1); O130:H6-E-ST405-CH37-27 (1); ONT:H6-E-ST405-CH37-27 (1)
			CTX-M-14 (8)	O7:H4-A-ST93-CH11-31 (1); O9:H4-A-ST615-CH7-34 (1); O101:H10-A-ST617-CH11-negative (1); O153:H19-C-ST58-CH4-25 (1); O91:H28-B1-ST1196-CH6-31 (1); O2:HNM-B2-ST141-CH52-14 (1); **O16:H5-B2-ST131-CH40-41 (1)**; O77:H18-E-ST106-CH35-47 (1)
			CTX-M-9 (1)	**O25b:H4-B2-ST131-CH40-22 (1)**
			SHV-12 (2)	O68:H21-B1-ST602-CH19-86 (1); ONT:HNM-A-ST10-CH11-54 (1)
2011	313	26 (8.3%)	CTX-M-15 (11)	**O25b:H4-B2-ST131-CH40-30 (11)**
			CTX-M-14 (8)	O7:H4-A-ST93-CH11-31 (1); O8:H4-C-ST88-CH4-39 (1); O9:H4-A-ST609-CH7-0 (1); O11:H9-C-ST1615-CH263-32 (1); O8:H7-B1-ST1642-CH4-31 (1); O23:H28-B1-ST156-CH29-38 (1); O153:H34-F-ST354-CH88-58 (1); ONT:H34-F-ST354-CH88-58 (1)
			CTX-M-1 (5)	ONT:HNM-A-ST1630- CH11-400 (1); O9:H4-A-ST1421-CH7-54 (1); O54:H21-B1-ST58-CH4-0 (1); ONT:H25-B1-ST641-CH6-479 (1); O153:H15-E- ST973-CH187-27 (1)
			SHV-12 (2)	**O25b:H4-B2-ST131-CH40-22 (1)**; O2:HNM-F-ST648-CH4-58 (1)
**Total**	**2.427**	**96 (4.0%)**		

*^*a*^The ST131 isolates are highlighted in bold. ^*b*^New ST: *adk*-10, *fumC*-11, *gyrB*-4, *icD*-8, *mdh*-492, *purA*-8 and *recA*-2.*

### ESBL Enzymes

**FIGURE 2:**
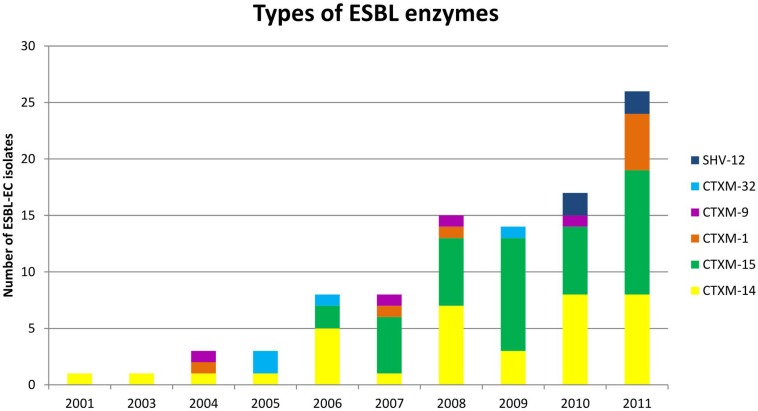
Distribution of extended-spectrum β-lactamase (ESBL) enzymes. Of the 96 ESBL-EC bloodstream isolates, 40 were positive for CTX-M-15, 36 for CTX-M-14, eight for CTX-M-1, and four for CTX-M-9, CTX-M-32, and SHV-12 each.

Of the 96 ESBL-EC isolates, 40 were positive for CTX-M-15, 36 for CTX-M-14, eight for CTX-M-1, and four for CTX-M-9, CTX-M-32, and SHV-12 each (Figure 2 and Table 1).

### Phylogenetic Groups

The commonest phylogenetic group was B2 (44 isolates), followed by B1 (18 isolates), A and E (11 isolates from each), F (six isolates), C (five isolates), and D (one isolate).

The majority of CTX-M-15-producing (87.5%, 35/40) isolates belonged to phylogenetic group B2. In contrast, only five (14%) of 36 CTX-M-14-producing isolates were assigned to this phylogenetic group (*P* < 0.001) (Table 1).

### Multilocus Sequence Types

The 96 ESBL-EC isolates belonged to 36 different STs (Table 1). The commonest was ST131 (41 isolates), followed by ST58 (four isolates), ST354 (four isolates), ST393 (four isolates), ST405 (four isolates), and ST359 (three isolates). Most CTX-M-15 isolates (87.5%, 35/40) were ST131, whereas the CTX-M-14 isolates belonged to 23 different STs, none of which accounted for >12% and only 3 (8.3%) of 36 isolates were ST131 (Table 1).

### Clonotypes

CH typing identified 40 clonotypes, with CH40-30 being the most prevalent, which was present in 35 ST131 CTX-M-15-producing isolates. Other prevalent clonotypes were: CH41-35 (three ST359 isolates), CH40-22 (three ST131 isolates), CH106-54 (four ST393 isolates), CH37-27 (four ST405 isolates), and CH88-58 (four ST354 isolates) (Table 1).

### Serotypes

The 96 ESBL-EC isolates belonged to 21 O serogroups and expressed 17 H flagellar antigens. However, 39 ST131 isolates belonged to serotype O25b:H4. Other prevalent serotypes were: O15:H1 (four ST393 isolates), O9:H4 (three CC46 isolates), O7:H4 (two ST93 isolates), O8:H7 (two ST1642 isolates), O101:H10 (two CC10 isolates), and O153:H34 (two ST354 isolates) (Table 1).

### Virulence Genes

The ST131 isolates showed a higher virulence score (range 11 to 21; mean 13.49) compared with the non-ST131 isolates (range 1 to 18; mean 8.31) (*P* < 0.001).

Fourteen virulence genes (*afa/draBC*, *afaFM955459*, *yfcV*, *sat*, *cnf1*, *iucD*, *iutA*, *fyuA*, *chuA*, *kpsM II*, *kpsM II-K2*, *malX*, *usp*, and *ompT*) were significantly associated with ST131 isolates, whereas only five virulence genes (*fimAv*_MT78_, *hlyF*, *vat*, *iroN*, *cvaC*, *iss*, and *traT*) were significantly associated with non-ST131 isolates.

Of the 96 ESBL-EC isolates, 65.6% were classified as ExPEC and 44.8% classified as UPEC. The prevalence of ExPEC (97.6 vs. 41.8%) (*P* < 0.001) and UPEC (90.2 vs. 10.9%) (*P* < 0.001) status were higher within ST131 isolates than within non-ST131 isolates (Table 2).

**TABLE 2:** Virulence genes in ST131 and non-ST131 bloodstream isolates.

**Gene(s)**	**Comment**	**Total isolates (*n* = 96)**	**ST131 isolates (*n* = 41)**	**Non-ST13 isolates (*n* = 55)**	***P*-value^a^ ST131 vs. non-ST131**
**Adhesins**					
*fimH*	D-mannose-specific adhesin, type 1 fimbriae	94 (97.9%)	40 (97.6%)	54 (98.1%)	
*fimAv*_*MT78*_	Fim A variant MT78 of type 1 fimbriae	10 (10.4%)	0	**10 (18.2%)**	0.003
*papAH*	P fimbriae operon. Major structural subunit	21 (21.9%)	10 (24.4%)	11 (20.0%)	
*papC*	P fimbriae operon. Pilus assembly	21 (21.9%)	10 (24.4%)	11 (20.0%)	
*papEF*	P fimbriae operon. Minor tip pilins.	22 (22.9%)	10 (24.4%)	12 (21.8%)	
*papG I*	P fimbriae operon. Allele I of *papG* gene	0	0	0	
*papG II*	P fimbriae operon. Allele II of *papG* gene	16 (16.7%)	9 (22.0%)	7 (12.7%)	
*papG III*	P fimbriae operon. Allele III of *papG* gene	2 (2.1%)	1 (2.4%)	1 (1.8%)	
*papG IV*	P fimbriae operon. Allele IV of *papG* gene	0	0	0	
*sfa/focDE*	*Sfa* (S fimbriae) and *foc* (F1C fimbriae) operons	1 (1.0%)	0	1 (1.8%)	
*afa/draBC*	Dr antigen-specific adhesin operons	27 (28.1%)	**27 (65.9%)**	0	< 0.001
*afaFM955459*	Operon afa specific for clone O25b-ST131	26 (27.1%)	**26 (63.4%)**	0	< 0.001
*yfcV*	Putative chaperone-usher fimbria	49 (51.0%)	**37 (90.2%)**	12 (21.8%)	< 0.001
**Toxins**					
*sat*	Secreted autotransporter toxin	45 (46.9%)	**37 (90.2%)**	8 (14.5%)	< 0.001
*cnf1*	Cytotoxic necrotizing factor 1	4 (4.2%)	**4 (9.8%)**	0	0.030
*hlyA*	α-hemolysin	5 (5.2%)	4 (9.8%)	1 (1.8%)	
*hlyF*	Hemolysin F	25 (26.0%)	4 (9.8%)	**21 (38.2%)**	0.001
*cdtB*	Cytolethal distending toxin	1 (1.0%)	1 (2.4%)	0	
*tsh*	Tsh (temperature-sensitive hemagglutinin) serine protease	10 (10.4%)	2 (4.9%)	8 (14.5%)	
*vat*	Vacuolating autotransporter toxin. Serine protease	6 (6.3%)	0	**6 (10.9%)**	0.031
**Iron uptake**					
*iucD*	Ferric aerobactin receptor	81 (84.4%)	**41 (100%)**	40 (72.7%)	< 0.001
*iutA*	Ferric aerobactin receptor	81 (84.4%)	**41 (100%)**	40 (72.7%)	< 0.001
*iroN*	Catecholate (salmochelin) siderophore receptor	21 (21.9%)	3 (7.3%)	**18 (32.7%)**	0.002
*fyuA*	*Yersinia* siderophore receptor	65 (67.7%)	**41 (100%)**	24 (43.6%)	< 0.001
*chuA*	Heme binding outer membrane	62 (64.6%)	**41 (100%)**	21 (38.2%)	< 0.001
**Capsule**					
*kpsM II*	Group II capsule	60 (62.5%)	**40 (97.6%)**	20 (36.4%)	< 0.001
*kpsM II-K2*	K2 variant of group II capsule	27 (28.1%)	**27 (65.9%)**	0	< 0.001
*kpsM II-K5*	K5 variant of group II capsule	29 (30.2%)	11 (26.8%)	18 (32.7%)	
*neuC-K1*	K1 antigen	4 (4.2%)	2 (4.9%)	2 (3.6%)	
*kpsM III:*	Group III capsule	3 (3.1%)	0	3 (5.5%)	
**Miscellaneous**					
*cvaC*	ColV (microcin V)	17 (17.7%)	3 (7.3%)	**14 (25.5%)**	0.018
*iss*	Increased serum survival (OMP)	19 (19.8%)	3 (7.3%)	**16 (29.1%)**	0.007
*traT*	Serum resistance-associated (OMP)	56 (58.3%)	18 (43.9%)	**38 (69.1%)**	0.012
*ibeA*	Invasion of brain endothelium	9 (9.4%)	3 (7.3%)	6 (10.9%)	
*malX*	Pathogenicity-associated island marker (PAI)	55 (57.3%)	**41 (100%)**	14 (25.5%)	< 0.001
*usp*	Uropathogenic-specific protein (bacteriocin)	48 (50.0%)	**41 (100%)**	7 (12.7%)	< 0.001
*ompT*	Outer membrane protein (protease) T	70 (72.9%)	**41 (100%)**	29 (52.7%)	< 0.001
**ExPEC status**	If positive for ≥2 of 5 markers, including *papAH* and/or *papC*, *sfa/focDE*, *afa/draBC*, *kpsM II* and *iutA*	63 (65.6%)	**40 (97.6%)**	23 (41.8%)	< 0.001
**UPEC status**	If positive for ≥3 of 4 markers, including *chuA*, *fyuA*, *vat* and *yfcV*.	43 (44.8%)	**37 (90.2%)**	6 (10.9%)	< 0.001

*^*a*^*P*-values were calculated using the Fisher’s exact test and are shown where *P* < 0.05. Significant differences are indicate in bold.*

### *H*30Rx Subclone and Virotypes of ST131 Isolates

The 35 ST131 O25b:H4 CTX-M-15-producing isolates belonged to the *H*30Rx subclone. Twenty-six of the 35 *H*30Rx isolates showed the virotype A, four isolates the virotype E and five isolates the virotype F. In contrast, the three isolates of *H*22 subclone showed the virotypes D2 (one isolate) and D4 (two isolates). Besides, one of the two isolates belonging to *H*41 subclone showed the virotype C3, while the other was non-typeable (NT).

**FIGURE 3:**
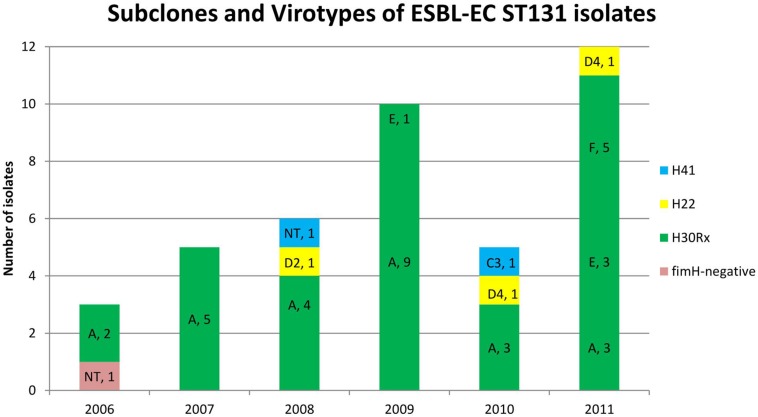
Distribution of subclones and virotypes of extended-spectrum β-lactamase-producing *E. coli* (ESBL-EC) ST131 bloodstream isolates. A drastic change in ST131 virotypes happened in 2011 due to the emergence of the virotypes E and F, which displaced virotype A.

In 2011, there was a very drastic change in the virotypes, due to the emergence of the virotypes E (*sat*, *papGII*, *cnf1*, *hlyA*, and *kpsMII-K5*) and F (*sat*, *papGII*, and *kpsMII-K5*) which have displaced the virotype A (*afa/draBC*, *afa* operon FM955459, *sat*, and *kpsMII-K2*). Thus 8 (66.7%) of the 12 ST131 isolates obtained in 2011 belonged to virotypes E and F vs. 1 (3.4%) of 29 ST131 isolates from 2006 to 2010 (*P* < 0.001). By contrast, while in the first period 23 (79.3%) of 29 ST131 isolates belonged to virotype A in the last year represented only 3 (25%) of 12 isolates (*P* = 0.002) (Figure 3).

### Macrorestriction Profiles by PFGE

Supplementary Figures S1, S2 show a dendrogram with the *XbaI* macrorestriction profiles of the ST131 and non-ST131 which showed a similarity of 61.3 and 50.2%, respectively. A high genetic diversity was detected among the 40 ST131 isolates, showing 31 different PFGE profiles. However, the isolates remained distributed within three virotype-specific clusters of 75% (four virotype E isolates), 84.2% (25 virotype A isolates) and 94.6% identity (five virotype F isolates). As expected, the 55 non-ST131 isolates showed even more heterogeneity, with 55 different profiles. However, the majority of isolates belonging to the same STs, clonotypes and O:H serotypes grouped together in the dendrogram.

## Discussion

The management of BSIs due to *E. coli* has been complicated due to antimicrobial resistance emergence, especially since 2000. A low proportion of the bacteraemia *E. coli* isolates recovered in our hospital between 1989 and 1992 were resistant to extended-spectrum cephalosporins (ESC) (0.5%) and fluoroquinolones (FQs) (1%), and none of the 213 *E. coli* isolates was multidrug resistant (MDR). However, a significant number turned out to be resistant (ESC 8%, FQs 31%, and MDR 16%) within 614 bacteraemia *E. coli* isolates obtained between 2010 and 2011 (unpublished data).

*Escherichia coli* BSIs are increasing in Europe and worldwide mainly due to the increased resistance to antibiotics, and especially to the expansion of high-risk clones such as ST131 (Tumbarello et al., 2010; de Kraker et al., 2013; Vihta et al., 2018). Since 2006, it is evident that the prevalence of ESBL-EC has raised in our hospital. This increase has been due to the spread of the multidrug-resistant *H*30Rx subclone associated with the production of CTX-M-15. Thus, the number of ESBL-EC isolates increased from 1.0% during 2000–2005 to 5.5% during 2006–2011. While during the first period 0% of the ESBL-EC isolates belonged to *H*30Rx subclone, in the second period this subclone represented 39.8%. A similar situation has been reported in other hospitals worldwide. Specifically, in a centralized Canadian region (Calgary), Peirano et al. (2012) investigated the prevalence and molecular characteristics of ESBL-EC bloodstream isolates obtained from the year 2000 to 2010 and found that these isolates increased significantly in the last years of the study (2% during 2000–2006 vs. 8% during 2007–2010). ST131 was first described in 2001, and its prevalence remained stable until 2006. However, since 2007, the prevalence of ST131 isolates increased significantly. Like in our study, most isolates from Canada produced either CTX-M-15 or CTX-M-14 (Peirano et al., 2012).

In the present study, while most of the 40 CTX-M-15-producing isolates belonged to ST131, the 36 CTX-M-14 positive isolates were distributed within 23 different STs. Similar results were observed by Merino et al. (2016) who reported a prevalence of 9.2% (39/425) of ESBL-EC among isolates causing bacteraemia of urinary origin in eight Spanish hospitals during 2010 and 2011. Of the 39 ESBL-EC isolates, 21 produced CTX-M-15 and 11 CTX-M-14. Fifteen STs were identified, but as in our study, the ST131 *H*30Rx subclone was predominant among CTX-M-15 isolates (20 of 21 isolates). Another seven STs (ST69, ST156, ST359, ST405, ST410, ST453, and ST609) reported by Merino et al. (2016) were also identified in the present study. However, the phylogroup distribution of our study differed from the one showed by Merino et al. (2016): A (11.5 vs. 7.7%), B1 (18.8 vs. 23.1%), B2 (45.8 vs. 53.8%), C (5.2 vs. 5.1%), D (1.0 vs. 10.3%), E (11.5 vs. 0%), and F (6.3 vs. 0%).

In our investigation, the CH typing identified 40 clonotypes, subdiving ST131 isolates in three subclones: CH40-30 (35 *H*30-Rx isolates of subclade C2), CH40-22 (three *H*22 isolates of clade B), and CH40-41 (two *H*41 isolates of clade A). Recently, Roer et al. (2018) presented a new Web tool for CH typing. To determine the resolution of it, 243 *E. coli* isolates were analyzed. Those isolates were resistant to third-generation cephalosporins and obtained from Danish patients with BSIs. A total of 48 different STs were identified, with ST131 the most common (50.2% of isolates; 95 CH40-30, 14 CH40-27, 11 CH40-41, one CH40-22, and one CH40-35). In addition to ST131, other 15 STs (ST23, ST58, ST69, ST88, ST93, ST95, ST141, ST345, ST354, ST393, ST405, ST410, ST453, ST617, and ST648) were identified in both studies. Thus, 72.9% of the Spanish isolates presented the same STs as 72.8% of the Danish isolates. Besides, the majority of the Spanish and Danish bloodstream isolates of the same ST displayed as well the same clonotype. Also, many of the STs found among our isolates were identified within the ESBL-EC isolates collected from BSIs in Japan (ST10, ST23, ST58, ST69, ST93, ST95, ST131, ST156, ST354, ST362, ST393, ST405, ST602, and ST648) (Matsumura et al., 2013), in Korea (ST10, ST69, ST95, ST131, ST354, ST393, ST405, ST410, ST453, ST617, ST648, and ST1642) (Kang et al., 2013), and in China (ST10, ST23, ST58, ST69, ST95, ST131, ST393, ST405, ST410, ST602, ST617, ST648, and ST1642) (Wang et al., 2016).

In Japan and other Asian countries, the CTX-M-27-producing C1/*H*30R-M27 and CTX-M-14-producing C1/*H*30R-nM27 subclades are more frequently isolated than the CTX-M-15-producing C2/*H*30Rx subclade (Matsumura et al., 2017b). We have not detected these two subclades within the collection of the study reported here (2000-2011). However, the epidemiologic situation seems to be changing in Spain since we have detected the subclade C1/*H*30R-M27 (five cases) within 92 ESBL-EC obtained from patients with urinary tract infections (84 cases) and other extraintestinal infections of our hospital in 2015, being the most frequent after the C2/*H*30Rx subclade (27 cases) (Flament-Simon et al., 2019).

The ST131 bloodstream isolates showed a higher virulence score than non-ST131 isolates. Furthermore, the prevalence of the ExPEC and UPEC status was also higher among the ST131 isolates than in the non-ST131 isolates, supporting the idea of a greater virulence potential of ST131, confirming the findings of previous studies (Blanco et al., 2011, 2013; Dahbi et al., 2014; Merino et al., 2016). However, the success of ST131 isolates cannot be explained exclusively by its high number of virulence genes. ST131 isolates are carriers of both virulence and resistance genes, which is very rare in classical extraintestinal pathogenic *E. coli* isolates (Nicolas-Chanoine et al., 2014). In addition, most ESBL-producing ST131 isolates are resistant to both extended-spectrum cephalosporins and fluoroquinolones (Colpan et al., 2013; Johnson et al., 2013). Diverse fitness cost associated with high-level resistance to fluoroquinolones was reported to contribute to the selection of the international clones of methicillin-resistant *Staphylococcus aureus*, *Clostridium difficile* ESBL-producing *Klebsiella pneumoniae* and *E. coli* (Fuzi et al., 2017). It is also important to highlight that the major reservoir of human extraintestinal pathogenic *E. coli* is the human digestive tract and that ST131 is the most competitive of the phylogenetic group B2 clones known to colonize the human digestive tract (Blanc et al., 2014; Nicolas-Chanoine et al., 2014). Recently, McNally et al. (2019) after analyzing the genome of 1,094 systematically sampled bacteremia ST131 isolates from the British Society for Antimicrobial Chemotherapy (BSAC) collection, found that clade C had accumulated a significantly elevated allelic diversity, particularly enriched for genes involved in anaerobic metabolism as well as other loci important for colonization of the human host by ExPEC.

In the present study, most of the isolates ST131 showed the virotype A. The international pulsotype L, which was responsible for the significant increase in BSIs in Calgary since 2007, also belonged to the virotype A and subclone *H*30Rx (Peirano et al., 2014). However, in many countries, the virotype C is the most frequent among ESBL-EC isolates belonging to *H*30Rx subclone (Olesen et al., 2014; Peirano et al., 2014). The isolates of virotypes E and F obtained during the last year (2011) of our study may possibly represent new sublineages of the ST131 clonal group.

A high genetic diversity was detected by PFGE. However, the ST131 isolates grouped in three specific clusters of virotypes A, E, and F suggesting a clonal basis for the virotypes (Blanco et al., 2013). In future studies it would be very interesting to determine the whole genome sequence of the ST131 isolates belonging to virotypes A, E and F to know if they really belong to different branches within the *H*30Rx C2 subclade.

In the present study we have not detected any carbapenem-resistant *E. coli*, but O25b:H4-ST131- *H*30-Rx isolates of virotype E and co-producing CTX-M-15 and OXA-48 were recently reported in another Spanish hospital (HUCA, Oviedo) near our region (de Toro et al., 2017).

The present study has certain limitations such as it was performed in a single hospital, non-ESBL ST131 isolates were not included, lack of whole genome sequencing (WGS) analysis or the fact that the last isolates were obtained during 2011.

## Conclusion

ST131 was the sequence type most frequently detected; moreover, the *H*30Rx subclone was responsible for the significant increase of ESBL-EC bloodstream isolates since 2006. In the year 2011 two new virotypes (E and F) of *H*30Rx subclone have emerged. Future molecular studies are needed to understand the dynamics of expansion of this successful high-risk subclone in order to prevent its spread and establish the importance of the two new virotypes.

## Data Availability

The raw data supporting the conclusions of this manuscript will be made available by the authors, without undue reservation, to any qualified researcher (Supplementary Table S2).

## Author Contributions

RM, SF-S, VG, MA, AM, CL, IG-M, DD-J, JEB, and MB undertook the laboratory work. JB conceived the concept for the research and designed the experiments. All authors provided critical input and contributed to the manuscript writing and approved its final version.

## Conflict of Interest Statement

The authors declare that the research was conducted in the absence of any commercial or financial relationships that could be construed as a potential conflict of interest.

## Footnotes

1 http://enterobase.warwick.ac.uk/species/ecoli/allele_st_search
