# Supplementary material for: Sequence Types, Clonotypes, Serotypes, and Virotypes of Extended-Spectrum β-Lactamase-Producing Escherichia coli Causing Bacteraemia in a Spanish Hospital Over a 12-Year Period (2000 to 2011)
Source: Front Microbiol. 2019 Jul 16;10:1530. doi: 10.3389/fmicb.2019.01530 (PMC6646471; doi:10.3389/fmicb.2019.01530)
Supplement: Supplementary file 1 [file Table_1.docx]

**SUPPLEMENTARY MATERIAL**

[https://www.frontiersin.org/articles/10.3389/fmicb.2019.01530/full#supplementary-material](https://www.frontiersin.org/articles/10.3389/fmicb.2019.01530/full#supplementary-material)

**Sequence types, Clonotypes, Serotypes and Virotypes of Extended-Spectrum β-Lactamase-Producing *Escherichia coli* Causing Bacteraemia in a Spanish Hospital over a 12-Year Period (2000 to 2011)**

**Rosalia Mamani^1^**^+^**, Saskia Camille Flament-Simon^1^**^+^**, Vanesa García^1^, Azucena Mora^1^, María Pilar Alonso^2^, Cecilia López^1^, Isidro García-Meniño^1^, Dafne Díaz-Jiménez^1^, Jesús E. Blanco^1^, Miguel Blanco^1^, and Jorge Blanco^1^***

^+^These authors made equal contributions to this study.

^1^Laboratorio de Referencia de *E. coli* (LREC), Departamento de Microbioloxía e Parasitoloxía, Facultade de Veterinaria, Universidade de Santiago de Compostela (USC), Lugo, Spain

^2^Unidade de Microbioloxía Clínica, Hospital Universitario Lucus Augusti (HULA), Lugo, Spain

***Corresponding author:** Jorge Blanco

**E-mail:** [jorge.blanco@usc.es](mailto:jorge.blanco@usc.es)

**Table S1.** Primers used in this study for PCR amplification of virulence genes.

**Table S2** (**Table 2.xlsx**) Characteristics of ESBL-producing *E. coli* bloodstream isolates. This table includes the raw data supporting the conclusions of this manuscript.

**Figure S1** shows a dendrogram with the *XbaI* macrorestriction profiles obtained by pulsed-field gel electrophoresis (PFGE) of the ST131 isolates with a similarity of 61.3%.

**Figure S2** shows a dendrogram with the XbaI macrorestriction profiles obtained by PFGE of the non-ST131 isolates with a similarity of 50.2%.

**Table S1.** Primers used in this study for PCR amplification of virulence genes

| **Gene** | **Function** | **Primer name** | **Sequence (5’-3’)** | **Reference** |
| --- | --- | --- | --- | --- |
| **Adhesins** | | | | |
| *fimH* | D-mannose-specific adhesin, type 1 fimbriae | fimH-F  fimH-R | TGCAGAACGGATAAGCCGTGG  gcagtcacctgccctccggta | Johnson and Stell, 2000 |
| *fimAv_MT78_* | Fim A variant MT78 of type 1 fimbriae | fimA215  fimA201 | ACTTTAGGATGAGTACTG  TCTGGCTGATACTACACC | Marc and Dho-Moulin, 1996 |
| *papAH* | P fimbriae (pyelonephritis-associated pilus) operon. Major structural subunit. Defines F antigen | papAH-F  papAH-R | ATGGCAGTGGTGTCTTTTGGTG  CGTCCCACCATACGTGCTCTTC | Johnson JR *et al*., 2015 |
| *papC* | P fimbriae operon. Pilus assembly | papC-F  papC-R | GTGGCAGTATGAGTAATGACCGTTA  ATATCCTTTCTGCAGGGATGCAATA | Johnson JR *et al*., 2015 |
| *papEF* | P fimbriae operon. Minor tip pilins. Connect PapG to PapA | papEF-F  papEF-R | GCAACAGCAACGCTGGTTGCATCAT  AGAGAGAGCCACTCTTATACGGACA | Yamamoto *et al*., 1995 |
| *papG I* | P fimbriae operon. Allele I of *papG* gene | Pap-I f  Pap-I r | TTAGCTGGATGGCACAATG  TTGTCCATGTATCCCATTCAT | Mora *et al*., 2013 |
| *papG II* | P fimbriae operon. Allele II of *papG* gene | Pap-II f  Pap-II r | GGGCATTGCTACGGTAACCTG  CGCTATTAATAGACAGATCACC | Mora *et al*., 2013 |
| *papG III* | P fimbriae operon. Allele III of *papG* gene | Pap-III f  Pap-III r | CGGCAACTTTAAGCTATGTG  TGTACCATCTCATCGTTGTCTC | Mora *et al*., 2013 |
| *papG IV* | P fimbriae operon. Allele IV of *papG* gene | Pap-IV f  Pap-IV r | TCCGAGAAGTACCCAAACCAC  GTCACAATAAATGGTAACAGCT | Blanco M.  This study |
| *sfa/focDE* | Central region of *sfa* (S fimbriae) and *foc* (F1C fimbriae) operons | sfa1  sfa2 | CTCCGGAGAACTGGGTGCATCTTAC  CGGAGGAGTAATTACAAACCTGGCA | Le Bouguenec *et al*., 1992 |
| *afa/draBC* | Dr antigen-specific adhesion operons (AFA, Dr, F1845) | afa1  afa2 | GCTGGGCAGCAAACTGATAACTCTC  CATCAAGCTGTTTGTTCGTCCGCCG | Le Bouguenec *et al*., 1992 |
| *afaFM955459* | Operon *afa* specific for clonal group O25b-ST131 | AFA-O25F  AFA-O25R | GAGTCACGGCAGTCGCGGCGG  TTCACCGGCGCACAGCCATCTCC | Blanco *et al.*, 2009 |
| *yfcV* | Putative chaperone-usher fimbria | yfcV forward  yfcV reverse | acatggagaccacgttcacc  gtaatctggaatgtggtcagg | Spurbeck *et al*., 2012 |
| **Toxins** | | | | |
| *sat* | Secreted autotransporter toxin | satF  satR | GCAGCTACCGCAATAGGAGGT  CATTCAGAGTACCGGGGCCTA | Johnson *et al*., 2003 |
| *cnf1* | Cytotoxic necrotizing factor 1 | cnf1-f2  cnf1-rc | CAGGAGGTACTTAGCAGCGT  TAATTTTGGGTTTGTATC | Mora *et al*., 2013 |
| *hlyA* | α-hemolysin | hly f  hly r | AACAAGGATAAGCACTGTTCTGGCT  ACCATATAAGCGGTCATTCCCGTCA | Yamamoto *et al*., 1995 |
| *hlyF* | Hemolysin F | hlyF f  hlyF r | TCGTTTAGGGTGCTTACCTTCAAC  TTTGGCGGTTTAGGCATTCC | Morales *et al*., 2004 |
| *cdtB* | Cytolethal distending toxin | cdt-s1  cdt-as1  cdt-s2  cdt-as2 | GAAAGTAAATGGAATATAAATGTCCG  AAATCACCAAGAATCATCCAGTTA  GAAAATAAATGGAACACACATGTCCG  AAATCTCCTGCAATCATCCAGTTA | Tóth *et al*., 2003 |
| *tsh* | Temperature-sensitive hemagglutinin. Serine protease | tsh03  tsh15 | GGTGGTGCACTGGAGTGG  AGTCCAGCGTGATAGTGG | Dozois *et al*., 2000 |
| *vat* | Vacuolating autotransporter toxin. Serine protease | vat forward  vat reverse | TCAGGACACGTTCAGGCATTCAGT  GGCCAGAACATTTGCTCCCTTGTT | Spurbeck *et al*., 2012 |
| **Iron uptake** | | | | |
| *iucD* | Aerobactin | Aer f  Aer r | TACCGGATTGTCATATGCAGACCGT  AATATCTTCCTCCAGTCCGGAGAAG | Yamamoto *et al*., 1995 |
| *iutA* | Ferric aerobactin receptor (iron uptake transport) | aer-851f  aer-1152r | GGCTGGACATCATGGGAACTGG  CGTCGGGAACGGGTAGAATCG | Johnson and Stell, 2000 |
| *iroN* | Catecholate (salmochelin) siderophore receptor | Ironec-f  Ironec-r | AAGTCAAAGCAGGGGTTGCCCG  GACGCCGACATTAAGACGCAG | Johnson *et al*., 2000 |
| *fyuA* | *Yersinia* siderophore receptor (ferric yersiniabactin uptake) | fyuA forward  fyuA reverse | GTAAACAATCTTCCCGCTCGGCAT  TGACGATTAACGAACCGGAAGGGA | Spurbeck *et al*., 2012 |
| *chuA* | Heme binding outer membrane | chuA-ITU-F  chuA-ITU-R | CTGAAACCATGACCGTTACG  TTGTAGTAACGCACTAAACC | Spurbeck *et al*., 2012 |
| **Capsule** | | | | |
| *kii-kpsM II* | Group II capsule | KpsII f  KpsII r | GCGCATTTGCTGATACTGTTG  CATCCAGACGATAAGCATGAGCA | Johnson and Stell, 2000 |
| *kpsM II-K2* | K2 variant of group II capsule | kpsII f  KpsII-K2r | GCGCATTTGCTGATACTGTTG  AGGTAGTTCAGACTCACACCT | Johnson and  O'Bryan 2004 |
| *kpsM II-K5* | K5 variant of group II capsule | K5 f  kpsII r | CAGTATCAGCAATCGTTCTGTA  CATCCAGACGATAAGCATGAGCA | Johnson and Stell, 2000 |
| *neuC-K1* | K1 variant of group II capsule | neu1  neu2 | AGGTGAAAAGCCTGGTAGTGTG  GGTGGTACATCCCGGGATGTC | Moulin-Schouleur *et al*. 2006 |
| *kpsM III* | Group III capsule | kps III f  kps III r | TCCTCTTGCTACTATTCCCCCT  AGGCGTATCCATCCCTCCTAAC | Johnson and Stell, 2000 |
| **Miscellaneous** | | | | |
| *cvaC* | ColV (microcin V); on plasmids with *iucD/iutA, iroN, sitA*, *iss* and *traT* | ColV-Cf  ColV-Cr | CACACACAAACGGGAGCTGTT  CTTCCCGCAGCATAGTTCCAT | Johnson and Stell, 2000 |
| *iss* | Increased serum survival (outer membrane protein) | is-f  is-r | CAGCAACCCGAACCACTTGATG  AGCATTGCCAGAGCGGCAGAA | Rodriguez-Siek *et al*., 2005 |
| *traT* | Serum resistance-associated (outer membrane protein) | TraT f  TraT r | GGTGTGGTGCGATGAGCACAG  CACGGTTCAGCCATCCCTGAG | Johnson and Stell, 2000 |
| *ibeA* | Invasion of brain endothelium | ibe10 f  ibe10 r | AGGCAGGTGTGCGCCGCGTAC  TGGTGCTCCGGCAAACCATGC | Johnson and Stell, 2000 |
| *malX* (PAI) | Pathogenicity-associated island marker (PAI) | MALX-F  MALX-R | GCATGAGCAGTGCGATACATCGC  AGGGCTGGGAAGTGGTTTAGCC | Mora *et al*., 2013 |
| *usp* | Uropathogenic-specific protein (bacteriocin) | usp-f  usp-r | ACATTCACGGCAAGCCTCAG  AGCGAGTTCCTGGTGAAAGC | Bauer *et al.,* 2002 |
| *ompT* | Outer membrane protein (protease) T | ompT-F  ompT-R | ATCTAGCCGAAGAAGGAGGC  CCCGGGTCATAGTGTTCATC | Johnson, *et al*. 2015 |

**References of Table S1**

**Bauer RJ, Zhang L, Foxman B, *et al.*.** Molecular epidemiology of 3 putative virulence genes for *Escherichia coli* urinary tract infection-*usp*, *iha*, and *iroN*(*E. coli*). *J Infect Dis* 2002; **185**: 1521-4.

**Blanco M, Alonso MP, Nicolas-Chanoine MH, *et al.*** Molecular epidemiology of *Escherichia coli* producing extended-spectrum β-lactamases in Lugo (Spain): dissemination of clone O25b:H4-ST131 producing CTX-M-15. *J Antimicrob Chemother* 2009; 63, 1135-41

**Dozois CM, Dho-Moulin M, Brée A, *et al.*** Relationship between the Tsh autotransporter and pathogenicity of avian *Escherichia coli* and localization and analysis of the Tsh genetic region. *Infect Immun* 2000; **68**: 4145-54.

**Johnson JR, Stell AL.** Extended virulence genotypes of *Escherichia coli* strains from patients with urosepsis in relation to phylogeny and host compromise. *J Infect Dis* 2000; **181**: 261-72. Erratum in: *J Infect Dis* 2000; **181**: 2122.

**Johnson JR, Russo TA, Tarr PI, *et al.*** Molecular epidemiological and phylogenetic associations of two novel putative virulence genes, *iha* and *iroN* (*E. coli*), among *Escherichia coli* isolates from patients with urosepsis. *Infect Immun* 2000; **68**: 3040-7.

**Johnson JR, Gajewski A, Lesse AJ, *et al*.** Extraintestinal pathogenic *Escherichia coli* as a cause of invasive nonurinary infections. *J Clin Microbiol* 2003; **41**: 5798-02.

**Johnson JR, O'Bryan TT.** Detection of the *Escherichia coli* group 2 polysaccharide capsule synthesis Gene *kpsM* by a rapid and specific PCR-based assay. *J Clin Microbio*l 2004; **42**: 1773-6.

**Johnson JR, Porter S, Johnston B, *et al*.** Host characteristics and bacterial traits predict experimental virulence for *Escherichia coli* bloodstream isolates from patients with urosepsis. *Open Forum Infect Dis* 2015; **2**(3):ofv083.

**Le Bouguenec C, Archambaud M, Labigne, A.** Rapid and specific detection of the *pap*, *afa*, and *sfa* adhesin-encoding operons in uropathogenic *Escherichia coli* strains by polymerase chain reaction. *J Clin Microbiol* 1992; **30**: 1189-93.

**Marc D, Dho-Moulin M.** Analysis of the *fim* cluster of an avian O2 strain of Escherichia coli: serogroup-specific sites within *fimA* and nucleotide sequence of *fimI*. *J Med Microbiol* 1996; **44**: 444-52.

**Moulin-Schouleur M, Schouler C, Tailliez P, *et al.*** Common virulence factors and genetic relationships between O18:K1:H7 *Escherichia coli* isolates of human and avian origin. *J Clin Microbiol* 2006; **44**: 3484-92.

**Mora A, Viso S, López *et al*.** Poultry as reservoir for extraintestinal pathogenic *Escherichia coli* O45:K1:H7-B2-ST95 in humans. *Vet Microbiol* 2013; **167**:506-12.

**Morales C, Lee MD, Hofacre C, *et al*.** Detection of a novel virulence gene and a *Salmonella* virulence homologue among *Escherichia coli* isolated from broiler chickens. *Foodborne Pathog Dis* 2004:**1**:160-5.

**Rodriguez-Siek KE, Giddings CW, Doetkott C, *et al.*** Characterizing the APEC pathotype. *Vet Res* 2005; **36**:241-56.

**Spurbeck RR, Dinh PC Jr, Walk ST, *et al*.** *Escherichia coli* isolates that carry *vat*, *fyuA*, *chuA*, and *yfcV* efficiently colonize the urinary tract. *Infect Immun* 2012; **80**:4115-22.

**Tóth I, Hérault F, Beutin L, *et al.*** Production of cytolethal distending toxins by pathogenic *Escherichia coli* strains isolated from human and animal sources: establishment of the existence of a new cdt variant (Type IV). *J Clin Microbiol* 2003; **41**: 4285-91.

**Yamamoto S, Terai A, Yuri K, *et al.*** Detection of urovirulence factors in *Escherichia coli* by multiplex polymerase chain reaction. *FEMS Immunol Med Microbiol* 1995; **12**: 85-90.


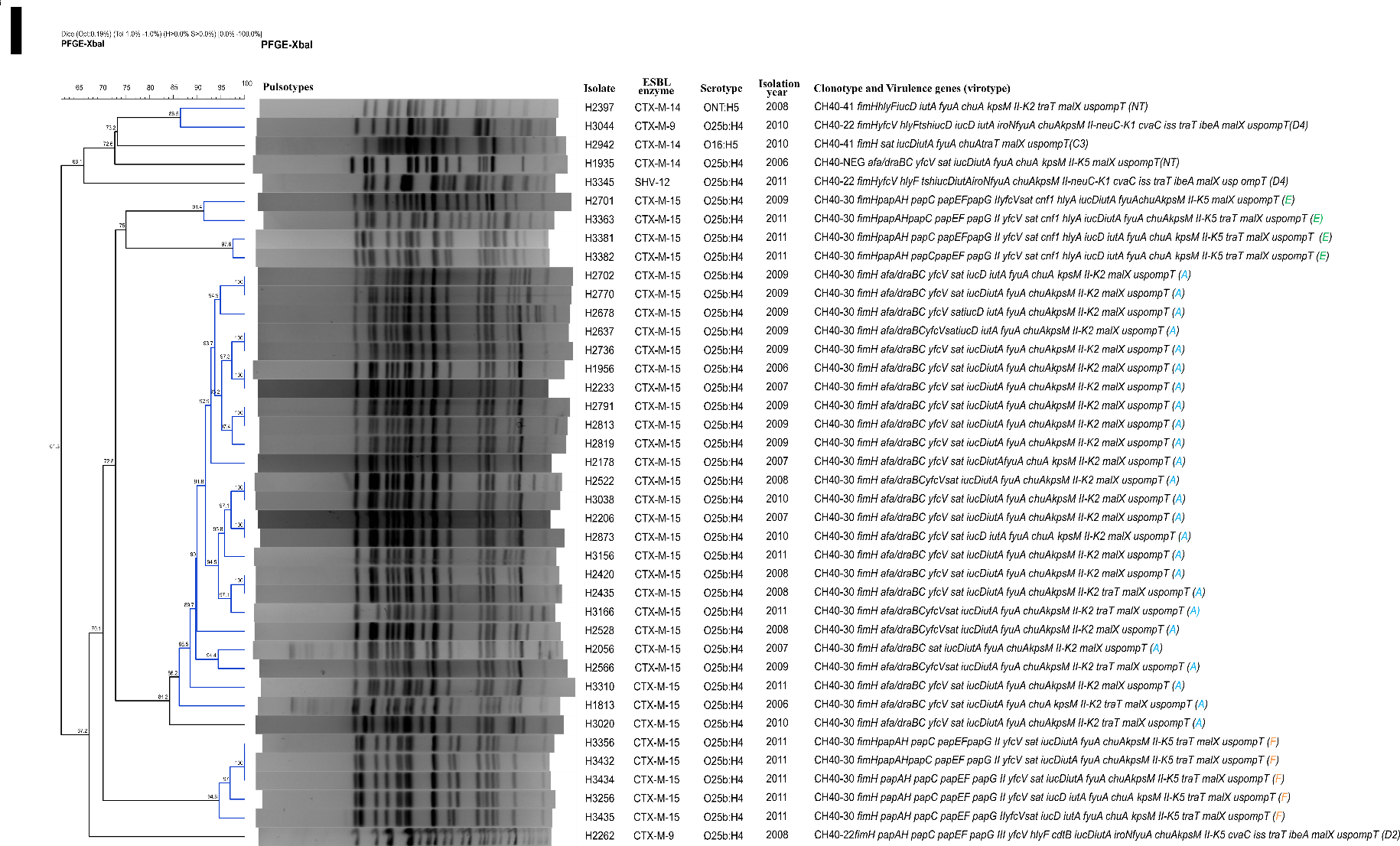


**Figure S1** (supplemental material) shows a dendrogram with the *XbaI* macrorestriction profiles obtained by pulsed-field gel electrophoresis (PFGE) of the ST131 isolates with a similarity of 61.3%. A high genetic diversity was detected, as 31 different PFGE profiles were obtained from the 40 ST131 isolates analysed. According to their PFGE similarities, the isolates were distributed among three virotype-specific clusters, defined at similarity levels of 75% (4 virotype E isolates), 84.2% (25 virotype A isolates) and 94.6% (5 virotype F isolates). The dendrogram was obtained with the UPGMA algorithm based on the DICE similarity coefficient and applying 1% of tolerance in the band position.


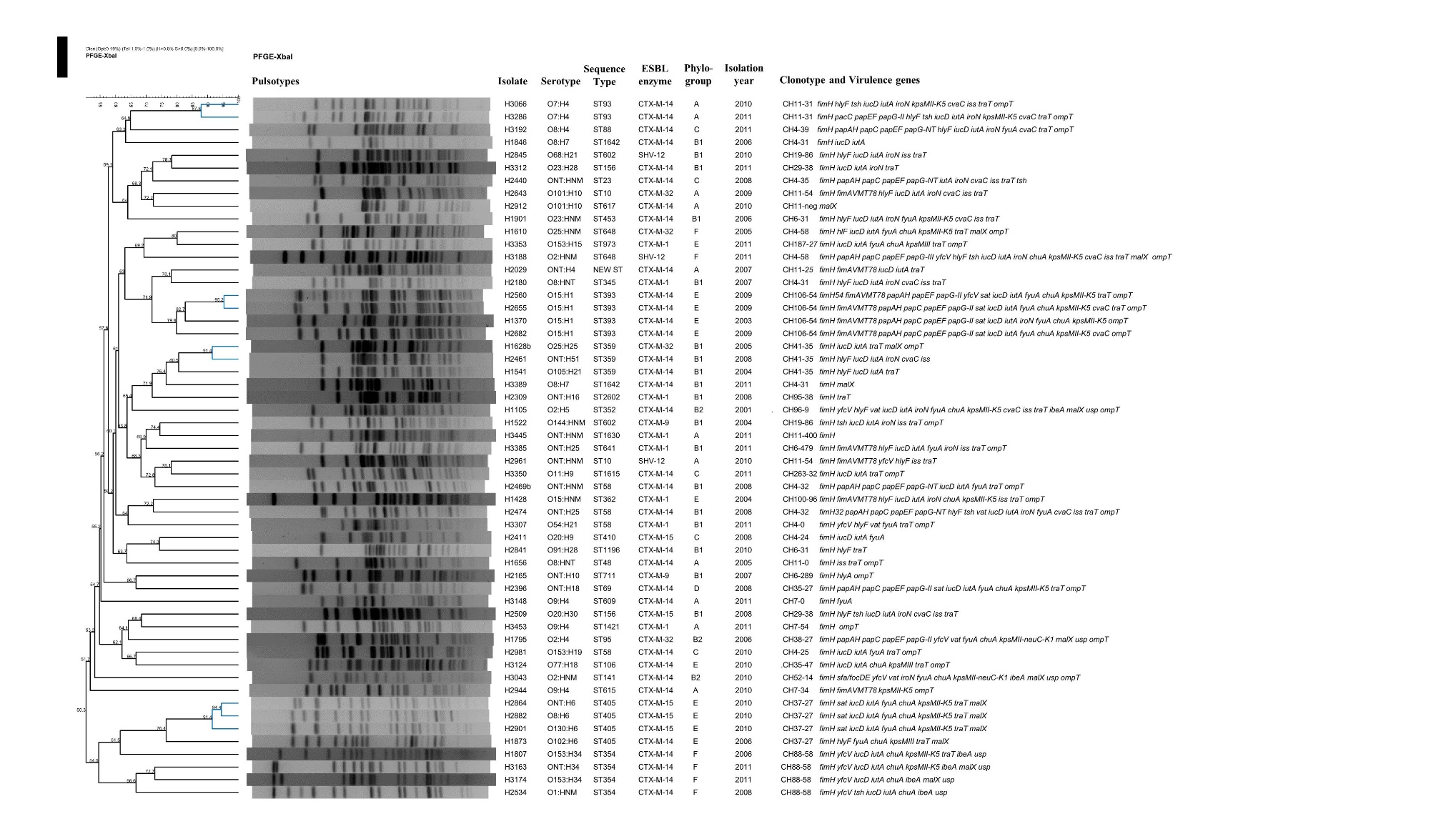


**Figure S2** (supplemental material) shows a dendrogram with the XbaI macrorestriction profiles obtained by PFGE of the non-ST131 isolates with a similarity of 50.2%. A very high genetic diversity was detected, as 55 different PFGE profiles were obtained from the 55 non-ST131 isolates analysed. However, the majority of isolates belonging to the same sequence types, clonotypes and O:H serotypes remained distributed in the same clusters. The dendrogram was obtained with the UPGMA algorithm based on the DICE similarity coefficient and applying 1% of tolerance in the band position.
